# A novel mutation in the *ACAN* gene in a family with autosomal dominant short stature and intervertebral disc disease

**DOI:** 10.1038/s41439-020-00132-8

**Published:** 2020-12-03

**Authors:** Noboru Uchida, Hironori Shibata, Gen Nishimura, Tomonobu Hasegawa

**Affiliations:** 1 Department of Pediatrics, Keio University School of Medicine, 35 Shinanomachi, Shinjuku-ku, Tokyo, Japan; 2 Center for Intractable Diseases, Saitama Medical University Hospital, 38 Morohongo, Moroyama-machi, Iruma-gun, Saitama, Japan

**Keywords:** Clinical genetics, Mutation

## Abstract

Heterozygous mutations in the *ACAN* gene have been reported in individuals with short stature and advanced bone age, with or without early-onset osteoarthritis and/or osteochondritis dissecans. We report a family with a phenotypic constellation carrying a novel mutation in the *ACAN* gene. The proband was a 7-year-old Japanese girl with short stature. Her mother and maternal grandmother also had short stature and intervertebral disc disease. We analyzed the *ACAN* gene in the family and identified a novel heterozygous mutation: c.4634delT, Leu1545Profs*11.

Aggrecan, encoded by the *ACAN* gene, is a cartilage-specific proteoglycan core protein that is a major structural component of the cartilage growth plate and intervertebral disc. Heterozygous *ACAN* mutations have been reported in individuals with short stature and advanced bone age, with or without early-onset osteoarthritis and/or osteochondritis dissecans (SSOAOD) (OMIM # 165800)^1–3^. The mutations identified to date are located throughout the protein, and more than half of them are premature truncating mutations^1–3^. Overall, the average adult height between individuals with truncating and missense mutations does not differ^1^. Except for an association with osteochondritis dissecans for two missense mutations located at the C-terminal C-type lectin domain, no definite genotype–phenotype correlation has been found^1,2^. The *ACAN* gene has a variable number tandem repeat (VNTR) at exon 12, which encodes the chondroitin sulfate attachment domain that dictates the length of the aggrecan core protein. We report a family with SSOAOD caused by a novel frameshift mutation at 234 bp from the 3′ end of the VNTR in *ACAN*.

The proband was a Japanese girl who is the third child of nonconsanguineous parents. She was delivered at 37 weeks of gestation with a birth length of 43.3 cm (−0.1 SD) and weight of 2350 g (−1.1 SD). Short stature had manifested since she was 1 year and 6-months old (Fig. 1A). She underwent endocrine assessment at 3 years of age, yielding normal results. She was referred to our hospital for further examinations at the age of 7 years and 8 months. Her height was 105.1 cm (−3.6 SD), weight was 19.4 kg (−1.4 SD), and arm span was 103 cm. She was noted to have a prominent jaw as a result of midface hypoplasia, a short left 4th toe, and broadening of the bilateral 5th toes. A skeletal survey showed no characteristic signs of skeletal dysplasia, other than minor changes of the digits, including mild shortening of the 4th and 5th metacarpals, shortening of the 1st distal phalanx (Fig. 1B), cone-shaped epiphyses of the left 1st and 4th metatarsals, and large epiphyses of the 5th distal phalanges (Fig. 1C). According to the Greulich and Pyle method, her bone age was 8 years and 10 months (Fig. 1B). The proband’s mother was 136.8 cm (−4.2 SD) tall, and her skeletal X-ray showed narrowed intervertebral spaces and endplate irregularities of the lower thoracic and upper lumbar vertebrae, indicative of early-onset intervertebral disc disease (Fig. 1D). The maternal grandmother was 134.4 cm (−4.7 SD) tall. She also had severe intervertebral disc disease and had undergone surgery for lumbar disc herniation (Fig. 1E). Other members of the family had an average height (Fig. 1F).

**Fig. 1:**
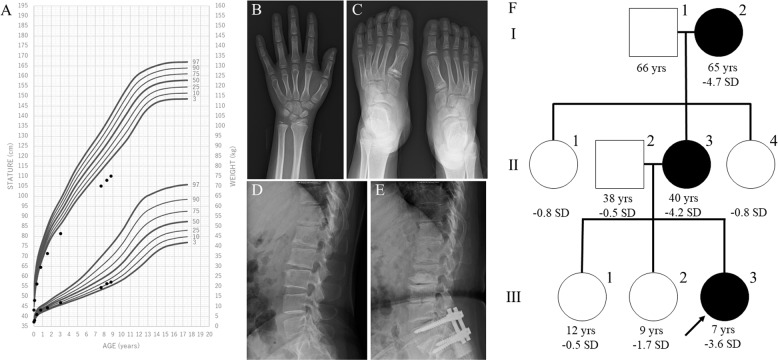
Clinical information of the family members. **A** Growth chart of the proband. Heights and weights are shown as black points. **B** X-ray of the proband’s left hand. Mild shortening of the 4th and 5th metacarpals and shortening of the 1st distal phalanx are shown. The bone age was interpreted as 8 years and 10 months using the Greulich and Pyle method. **C** X-ray of the proband’s feet. Cone-shaped epiphyses of the left 1st and 4th metatarsals and large epiphyses of the 5th distal phalanges are shown. **D**, **E** Lumbar spine X-rays of the proband’s mother and maternal grandmother. Narrowed intervertebral spaces and endplate irregularities are shown. **F** Pedigree of the studied family. The arrow indicates the proband (III-3). The family members with short stature carrying ACAN mutations are indicated with filled symbols. Ages and heights in standard division score are reported below each symbol.

After genetic counseling, we obtained informed consent from the family. The study was approved by the Ethics Committee of the Keio University School of Medicine. We extracted genomic DNA from peripheral blood samples of affected members of the family and amplified *ACAN* (NM_013227.3) coding exons and flanking introns, except for the regions flanking the VNTR in exon 12, as described previously^4–6^. To cover the regions flanking the VNTR, we designed additional sets of primers and performed polymerase chain reaction (details are provided in Supplementary tables). We also performed direct sequencing of the amplified products. DNA fragment sizes of the VNTR indicated 27 repeats in both the proband and her mother and 28 in the grandmother, which are common repeat numbers found in healthy adults^7,8^. By sequencing each side of the VNTR, we identified a novel heterozygous variant (c.4634delT, p.(Leu1545Profs*11)) at 234 bp from the 3′ end of the VNTR in the proband, mother, and maternal grandmother (Fig. 2). Genetic testing of other members was not permitted. This variant was not found in the Human Genetic Variation Database (HGVD; http://www.hgvd.genome.med.kyoto-u.ac.jp) or Genome Aggregation Database (gnomAD; https://gnomad.broadinstitute.org/). We conclude that this variant is a novel pathogenic mutation predicted to cause premature termination of translation and nonsense-mediated mRNA decay, leading to haploinsufficiency of *ACAN*.

**Fig. 2:**
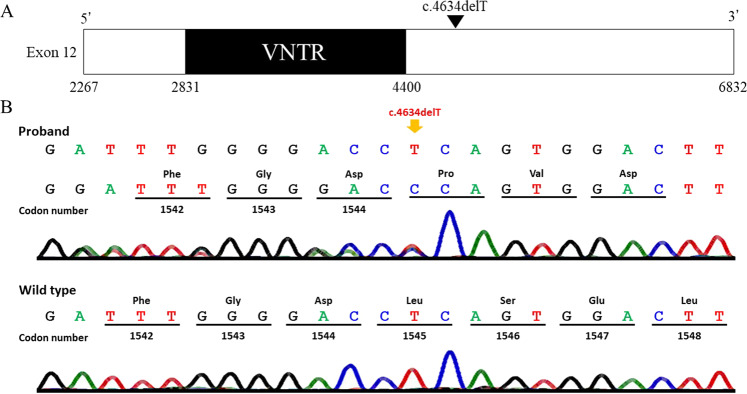
Exon 12 of the ACAN gene. **A** The black area on the exon 12 denotes various numbers tandem repeat. Variant c.4634delT (black triangle) is reported in this study. **B** Partial sequence (reverse complemented). The upper panel shows a chromatogram of the proband that had a heterozygous mutation, c.4634delT, Leu1545Profs*11, which is denoted by an arrow. The lower panel shows a chromatogram of the wild-type sequence.

The diagnosis of SSOAOD in this family is convincing. Clinically, some points are noteworthy. First, the short stature showed an autosomal dominant inheritance. Second, the proband had advanced bone ages of 1 year and 2 months compared to her chronological age, though not all previous patients with SSOAOD showed advanced bone age^1^. Third, the mother and grandmother had intervertebral disc diseases, consistent with a previous report by Dateki indicating that several cases of SSOAOD have an early onset and multiple intervertebral disc diseases^3^. Fourth, the proband had midface hypoplasia, a short left 4th toe and broad 5th toes. There have been reports on individuals with SSOAOD exhibiting midface hypoplasia and shortening or broadening of digits^1–3^. The phenotype of the proband suggested that mild dysmorphic findings may constitute syndromic components of *ACAN* mutations. SSOAOD should be considered in the differential diagnosis of children with autosomal dominant short stature, particularly in cases in which there is advanced bone age or a family history of intervertebral disc disease.

In conclusion, we identified a novel heterozygous *ACAN* mutation. Our report provides further evidence that *ACAN* mutations are associated with autosomal dominant short stature with intervertebral disc disease.

## Supplementary information

Supplementary Table 1: Additional sets of primers used in this study

Supplementary Table 2: PCR conditions using primers New 1 and New 2

## Data Availability

The relevant data from this Data Report are hosted at the Human Genome Variation Database at 10.6084/m9.figshare.hgv.2948.
