# Supplementary material for: A novel mutation in the ACAN gene in a family with autosomal dominant short stature and intervertebral disc disease
Source: Hum Genome Var. 2020 Dec 3;7:44. doi: 10.1038/s41439-020-00132-8 (PMC7712780; doi:10.1038/s41439-020-00132-8)
Supplement: Supplementary file 1 — Supplementary Table 1: Additional sets of primers used in this study [file 41439_2020_132_MOESM1_ESM.docx]

| **Primer** | | **Primer sequence (5’-3’)** |
| --- | --- | --- |
| **New 1** | **Forward** | CCTCAGCAGGCTCCCTTC |
|  | **Reverse** | CCGCTAAGCTCAGTCACTCC |
| **New 2** | **Forward** | CAGGCAGTGGAGATGTTTCA |
|  | **Reverse** | CAGTTTGCCCAAGTCCAAGT |
