# Supplementary material for: A novel mutation in the ACAN gene in a family with autosomal dominant short stature and intervertebral disc disease
Source: Hum Genome Var. 2020 Dec 3;7:44. doi: 10.1038/s41439-020-00132-8 (PMC7712780; doi:10.1038/s41439-020-00132-8)
Supplement: Supplementary file 2 — Supplementary Table 2: PCR conditions using primers New 1 and New 2 [file 41439_2020_132_MOESM2_ESM.docx]

| **No** | **PCR Steps** | **New 1** | **New 2** |
| --- | --- | --- | --- |
| 1) | Initial denaturation | 95°C for 5 min | 94°C for 2 min |
| 2) | Denaturation | 95°C for 30 sec | 98°C for 10 sec |
| 3) | Annealing | 60°C for 30 sec | 66°C for 30 sec |
| 4) | Extension | 72°C for 30 sec | 68°C for 30 sec |
| 5) | Total number of cycles. | 35cycles | 30 cycles |
| 6) | Final extension | 72°C for 5 min | 68°C for 5 min |
| Polymerase used in PCR | | AmpliTaq Gold 360  (Thermo　Fisher Scientific, Massachusetts, USA) | KOD FX Neo  (TOYOBO, Osaka, Japan) |

PCR, polymerase chain reaction
